# Observation of Fast Low‐Temperature Oxygen Ion Conduction in CeO_2_/β"‐Al_2_O_3_ Heterostructure

**DOI:** 10.1002/advs.202401130

**Published:** 2024-07-21

**Authors:** Yingbo Zhang, Decai Zhu, Zhonglong Zhao, Jiamei Liu, Yuzhao Ouyang, Jiangyu Yu, Zhongqing Liu, Xixi Bai, Nan Wang, Lin Zhuang, Wuming Liu, Chengjun Zhu

**Affiliations:** ^1^ Key Laboratory of Semiconductor Photovoltaic Technology and Energy Materials of Inner Mongolia Autonomous Region School of Physical Science and Technology Inner Mongolia University 235 West Daxue Street Hohhot Inner Mongolia 010021 P. R. China; ^2^ State Key Laboratory of Optoelectronic Materials and Technologies School of Physics Sun Yat‐Sen University Guangzhou 510275 P. R. China; ^3^ Beijing National Laboratory for Condensed Matter Physics Institute of Physics Chinese Academy of Sciences Beijing 100190 P. R. China

**Keywords:** heterostructure composite electrolyte, ionic conduction, local electric field, solid oxide fuel cell, thermal melting

## Abstract

Semiconductor ion fuel cells (SIFCs) have demonstrated impressive ionic conductivity and efficient power generation at temperatures below 600 °C. However, the lack of understanding of the ionic conduction mechanisms associated with composite electrolytes has impeded the advancement of SIFCs toward lower operating temperatures. In this study, a CeO_2_/β″‐Al_2_O_3_ heterostructure electrolyte is introduced, incorporating β″‐Al_2_O_3_ and leveraging the local electric field (LEF) as well as the manipulation of the melting point temperature of carbonate/hydroxide (C/H) by Na^+^ and Mg^2+^ from β″‐Al_2_O_3_. This design successfully maintains swift interfacial conduction of oxygen ions at 350 °C. Consequently, the fuel cell device achieved an exceptional ionic conductivity of 0.019 S/cm and a power output of 85.9 mW/cm^2^ at 350 °C. The system attained a peak power density of 1 W/cm^2^ with an ultra‐high ionic conductivity of 0.197 S/cm at 550 °C. The results indicate that through engineering the LEF and incorporating the lower melting point C/H, there approach effectively observed oxygen ion transport at low temperatures (350 °C), effectively overcoming the issue of cell failure at temperatures below 419 °C. This study presents a promising methodology for further developing high‐performance semiconductor ion fuel cells in the low temperature range of 300–600 °C.

## Graphical abstract

This study observes rapid oxygen ion conduction in CeO_2_/β″‐Al_2_O_3_ heterostructures at low temperatures 350–550 °C. This fast ion transport at heterointerfaces results from the synergistic effects of thermal melting and localized electric fields, offering a theoretical basis for advancing electrolyte development in SIFCs to achieve enhanced performance at reduced operating temperatures.

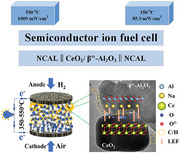

## 1 Introduction

Solid oxide fuel cells (SOFCs) are all‐solid‐state green energy conversion devices that have the characteristics of high efficiency and environmental protection.**^[^** ^1^ **^–^** ^3^ **^]^** The high operating temperature (700–1000 °C) of traditional SOFCs can achieve sufficient ion conductivity and quick fuel oxidation kinetics, but it also leads to many problems in terms of the high system cost and high corrosion rate of the fuel cell stack system.**^[^** ^4^ **^–^** ^6^ **^]^** However, due to the thermal activation nature of fuel cell work processes, directly lowering the operating temperature of SOFCs will significantly reduce the electrochemical performance of fuel cells.**^[^** ^7^ **^–^** ^9^ **^]^** Therefore, solving the problem of the poor ionic conductivity of SOFC electrolytes at relatively low temperatures has become the key to their commercialization. In recent years, the SIFCs using Ni_0.8_Co_0.15_Al_0.05_LiO_2_ (NCAL) as electrodes and heterostructure composite ionic conduction materials as electrolytes have provided a solution to improve electrochemical performance and ionic conductivity at temperatures below 600 °C, which has become a research hotspot in low‐temperature (LT) SOFCs.**^[^** ^10^ **^–^** ^15^ **^]^** In this approach, numerous studies have focused on developing advanced ion conductors by using nanocomposite electrolytes and adjusting the band bending/alignment effects.**^[^** ^16^ **^–^** ^20^ **^]^** Heterostructures have been acknowledged as a practical design strategy for swift ion transport materials both in terms of deployment and construction. Generally, when two semiconductor materials with different energy band levels are in contact, the offset of the conduction band and valence band is induced to obtain a continuous Fermi level, and the charge redistribution on the interface forms a space charge region. The electrostatic force of the built‐in electric field contributes to the electron blocking and ion accelerated transportation processes of the electrolyte in fuel cells. Meanwhile, our previous work has also verified that the heterostructure composite electrolytes constructed by introducing insulator (amorphous alumina, a‐Al_2_O_3_) into semiconductor (CeO_2_) also can exhibit high ion conductivity and promising application prospects. In which, a‐Al_2_O_3_ not only possesses thermoelectric and dielectric properties that promote battery stability, but also the introduction of insulators in heterostructures can form a wider depletion layer and higher potential barrier. More electron accumulation will exist near the heterointerface of the two‐phase materials, which will further act to achieve faster charge separation, thereby enabling the battery to demonstrate higher electrochemical performance.^[^ ^37^ ^]^

Notably, the performance of the SIFC rapidly decays at temperatures below 450 °C; thus, the operating temperature range of the SIFC is always between 450 and 600 °C. To date, the ion transport mechanism that determines the minimum operating temperature of SIFC has been partially explored. Research has shown that LiOH/Li_2_CO_3_ complexes can form at the NCAL anode of SIFCs and then diffuse into the electrolyte through capillary action. The LiOH/Li_2_CO_3_ composite exists in a molten state at temperatures above 419 °C,**^[^** ^21^ **^]^** and the amorphous layer formed by LiOH/Li_2_CO_3_ on the surface of electrolyte particles establishes interface conduction, which can significantly improve ion conduction and fuel cell performance.**^[^** ^22^ **^–^** ^25^ **^]^** When the temperature falls below 419 °C, the LiOH/Li_2_CO_3_ composite gradually solidifies, resulting in a significant decrease in oxygen ionic conductivity then reduction in fuel cell performance and loss of operational capacity.

Herein, this study aims to address the challenge of insufficient oxygen ionic conductivity in SIFCs, which leads to interface conduction failure at temperatures below 419 °C. To overcome this issue, we propose a novel design of a CeO_2_/β″‐Al_2_O_3_ heterostructure composite electrolyte and investigate the mechanism of oxygen ion transport within the CeO_2_/β″‐Al_2_O_3_ heterointerface. β″‐Al_2_O_3_ (Na_1.67_Mg_0.67_Al_10.33_O_17_) is a wide bandgap insulating material composed of basic units of spinel blocks and conducting planes. Na^+^ ions provide the 2D conducting planes distributed between the spinel blocks, while Mg^2+^ is introduced to stabilize the evaporation loss of Na^+^ at high temperatures. β″‐Al_2_O_3_ exhibits high sodium ion conductivity by diffusing Na^+^ ions within the 2D conducting planes separated by spinel blocks, and its high‐temperature stability and chemical inertness make it widely used in the preparation of ceramics, high‐temperature insulation materials, and electrochemical devices.^[^ ^27^, ^29^, ^50^, ^51^ ^]^ CeO_2_ is an n‐type semiconductor with a fluorite structure, which has been widely used and studied in various energy utilization fields such as fuel cells.^[^ ^52^, ^53^ ^]^ Its lattice structure has high defects, providing channels for oxygen ions to move. In addition, CeO_2_ usually has good compatibility with composite materials and can form stable interfaces.^[^ ^37^ ^]^ Therefore, in the design of composite electrolytes, CeO_2_ is frequently selected as a composite matrix to ensure electrolyte bulk ion conductivity while enhancing interface oxygen ion transport. By forming a heterostructure composite electrolyte with CeO_2_ and β″‐Al_2_O_3_, we achieved higher electrochemical performance and a lower operating temperature for SIFCs. As expected, by utilizing the accelerated transport of oxygen ions at heterogeneous structure interfaces, SIFCs using β″‐Al_2_O_3_/CeO_2_ electrolytes provide a peak power density of 1 W cm^−2^ at 550 °C, far exceeding that of pure CeO_2_ electrolytes. SIFCs using β″‐Al2O_3_/CeO_2_ as the electrolyte exhibit durability of ≈50 h without the influence of short‐circuit problems. Experimentally measured band information indicates that heterogeneous structures have higher barriers due to the significant difference in band gaps, effectively inhibiting electron transport. DFT calculations demonstrate that compared to CeO_2_, β″‐Al_2_O_3_/CeO_2_ exhibits a longer oxygen ion adsorption distance, indicating weaker bonding of oxygen ions at the heterostructure interface and therefore higher oxygen ion conductivity. Additionally, due to the presence of Na and Mg elements in β″‐Al_2_O_3_, C/H complexes of Na, Mg, and Li elements are formed within the electrolyte during the operation of the fuel cell. Experimental evidence has shown that the melting temperature of these complexes occurs at 353.5 °C. This is significantly lower than the melting point temperature of the Li_2_CO_3_/LiOH complex in traditional SIFCs (419 °C). The lower melting point temperature aims to ensure rapid oxygen ion transport at heterogeneous interfaces, allowing the fuel cell to function normally at 350 °C. Therefore, our research successfully observed fast oxygen ion conductivity at low temperatures.

## 2 Results and Discussion

### 2.1 Crystal Structure and Microstructure

**Figure 1:**
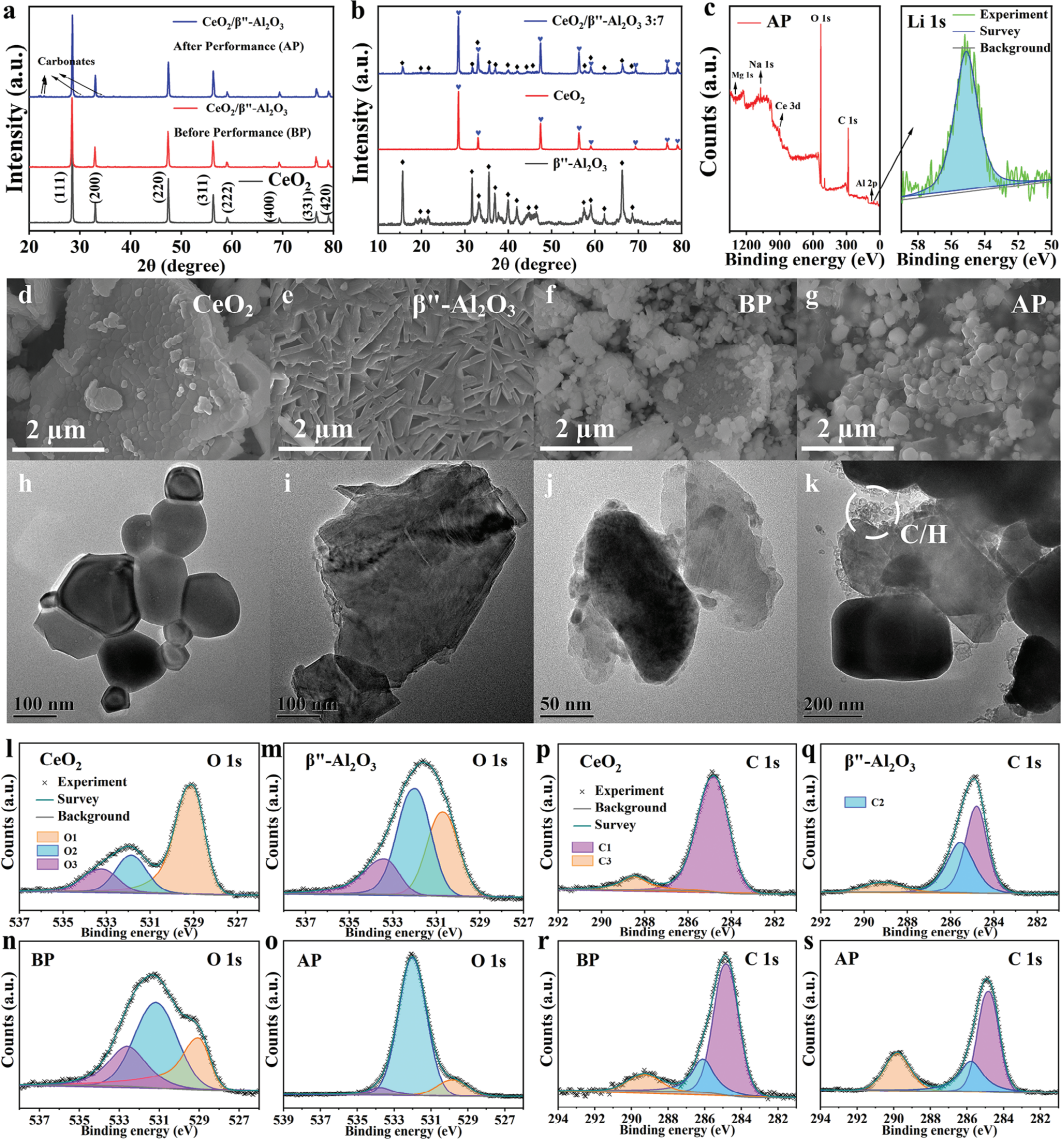
Characterizations of CeO_2_, β″‐Al_2_O_3_ and CeO_2_/β″‐Al_2_O_3_. a) X‐ray diffraction (XRD) patterns of pure CeO_2_ and the CeO_2_/β″‐Al_2_O_3_ 7:3 powders after performance (AP) and before performance (BP). b) XRD patterns of pure CeO_2_/β″‐Al_2_O_3_ 3:7 powders, CeO_2_ and pure β″‐Al_2_O_3_. c) Full spectrum and Li 1s X‐ray photoelectron spectra (XPS) spectrum in AP powder. Scanning electron microscopy (SEM) and transmission electron microscopy (TEM) images of d,h) CeO_2_, e,i) β″‐Al_2_O_3_, f,j) BP and g,k) AP, respectively. i–o) O 1s XPS spectrum and p–s) C 1s XPS spectrum of CeO_2_, β″‐Al_2_O_3_, BP and AP powder, respectively.

Figure S1a (Supporting Information) shows the phase structures of β″‐Al_2_O_3_ powders calcined at different temperatures. After quenching, the diffraction peak intensity and crystallinity of β″‐Al_2_O_3_ improved with increasing sintering temperature. Eventually, at 1400 °C, β″‐Al_2_O_3_ with excellent crystallinity was obtained for synthesizing composite electrolyte materials.**^[^** ^26^ **^–^** ^29^ **^]^** **Figure** **1**a presents the XRD patterns of synthesized CeO_2_ and CeO_2_/β″‐Al_2_O_3_ before electrochemical performance (BP) and after performance (AP). In these spectra, the main diffraction peaks were mainly attributed to the cubic fluorite structure (PDF No. 34–0394). 2θ = 28.555, 33.082, 47.479, 56.335, 59.087, 69.402, 76.700, and 79.070° corresponded to the (111), (200), (220), (311), (222), (400), (331), and (420) lattice planes, respectively. The diffraction peaks of metal carbonates are readily apparent in the AP powder (Figure S1b, Supporting Information), suggesting the formation of carbonates within the composite electrolyte after fuel cell test.**^[^** ^23^ **^,^** ^30^ **^]^** The XRD pattern of 3CeO_2_/7β″‐Al_2_O_3_ shown in Figure 1b clearly showed the superposition of the diffraction peaks of CeO_2_ and β″‐Al_2_O_3_, with no second or impurity phase in these patterns. These results indicated that stable CeO_2_/β″‐Al_2_O_3_ heterostructure composite materials were formed, and no chemical reaction occurred between the two materials. Furthermore, the microstructure of the four powders was observed using scanning electron microscopy (SEM) and transmission electron microscopy (TEM). As shown in Figure 1d,h, spherical‐like CeO_2_ particles exhibited clear grain boundaries, with a size distribution of ≈50–200 nm. From Figure 1e,i, β″‐Al_2_O_3_ had an irregular block shape with particle sizes ranging from 0.05–0.7 µm.**^[^** ^28^ **^]^** For the as‐prepared BP powder, a smaller β″‐Al_2_O_3_ uniformly coated on the CeO_2_ leads to the formation of a massive heterointerface within the composite electrolyte (Figure 1f,g). Subsequently, to further investigate the elemental distribution of the heterostructure composite materials, EDS mappings of BP are presented in Figure S2 (Supporting Information); here, Na, Mg, Al, Ce, and O are uniformly distributed over the entire region. The result indicates that β″‐Al_2_O_3_ and CeO_2_ display a consistent and mutually supportive uniform distribution within the composite material, and a widely existed interface conductive network favorable for oxygen ion transport is established between CeO_2_ and β″‐Al_2_O_3_.**^[^** ^31^ **^,^** ^32^ **^]^** In addition, from Figure 1g, there is a gray adhesive cover on the surface of the sample, which causes the spacing of the AP powder to be tightly linked. From Figure 1k, a certain amount of small particles became coated around the interface between CeO_2_ and β″‐Al_2_O_3_, and these substances are reported to be carbonates and hydroxides.**^[^** ^33^ **^]^**

### 2.2 X‐Ray Photoelectron Spectroscopy (XPS) Analysis

XPS analysis was used to detect the substances and oxygen species present in the electrolyte powder before and after electrochemical testing. Figure 1l,o show the fitted results of the O 1s core‐level spectra for the CeO_2_, β″‐Al_2_O_3_, BP and AP samples. All spectra have underwent a C‐peak correction, and the fitting background used the Shirley type. The fit could be divided into three binding peaks located at ≈(528–530 eV), (531–532 eV), and (532–534 eV), labeled O1, O2, and O3, respectively. The O1 peak belonged to lattice oxygen, the O2 peak was assigned to chemisorbed oxygen species adsorbed on the oxygen vacancies, and the O3 peak was attributed to surface oxide defects.**^[^** ^18^ **^,^** ^34^ **^,^** ^35^ **^]^** From Figure 1n, compared with pure CeO_2_, the BP sample showed a negative shift of O2 and O3 peaks; this shift was caused by the electronic density redistribution in the CeO_2_/β″‐Al_2_O_3_ heterostructure.**^[^** ^36^ **^]^** In addition, the BP/AP samples possessed a much higher relative ratio of (O2+O3)/O1, and the relative content of O2+O3 was positively correlated with the oxygen vacancy concentration these loosely bound oxygen species were easily released at the operating temperature of fuel cells,**^[^** ^37^ **^]^** and a large amount of oxygen vacancies were formed on the heterostructure composite of CeO_2_ and β″‐Al_2_O_3_, which could improve the oxygen ionic conductivity of the composite electrolyte. Moreover, as shown in Figure 1o, as the proportion of O2 in the AP sample significantly increased, the O1 and O3 peaks almost disappeared. This occurred because after fuel cell testing, the physically surface oxide defects O3 were converted into chemisorbed oxygen O2 (CO_3_^2−^ and ─OH).

Figure 1p,s shows the detailed C 1s XPS spectra results. The C1 fitting peak located at 284.8 eV is attributed to the C─C bands,**^[^** ^48^ **^]^** the C2 peak located at ≈286 eV is attributed to the C─O bands,**^[^** ^49^ **^]^** and the C3 peak located at 288–290 eV represents CO_3_^2−^ in the metal carbonate.**^[^** ^38^ **^]^** The relative content of the C3 peak in the AP sample is significantly higher than that in the BP sample, which confirms that carbonate infiltration and retention occur during the fuel cell testing process. Figure 1c shows the full spectrum and Li 1s XPS spectrum of the AP powder. The peak at 55.1 eV is attributed to Li^+^ from in the Li 1s spectrum. The characteristic peaks of the elements Li, Mg, Na, Ce, and Al can be easily observed in the survey spectra. Therefore, the C/H composites are present in the AP sample, and when the fuel cell is in operation, they work together with the interface effect of the heterostructure to enhance the electrochemical performance and reduce the operatable temperature of the fuel cells.

### 2.3 Fuel Cell Performance and Electrochemical Impedance Spectroscopy (EIS) Analysis

**Figure 2:**
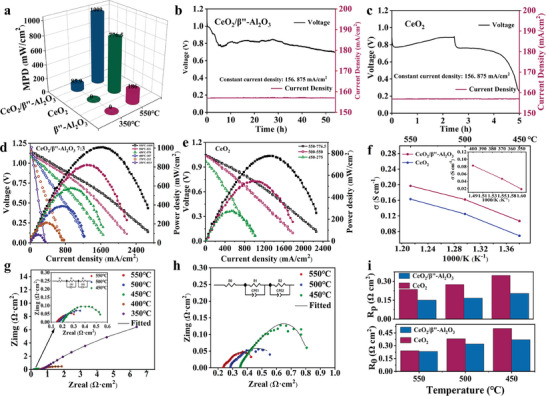
Performance and stability of CeO_2_/β″‐Al_2_O_3_ and CeO_2_ SIFCs. a) Power output of CeO_2_, β″‐Al_2_O_3_, and CeO_2_/β″‐Al_2_O_3_ devices operated at 550 and 350 °C. Stability test of the b) CeO_2_/β″‐Al_2_O_3_ and c) CeO_2_ fuel cells in a H_2_/air environment at 550 °C. d) Electrochemical performance of the CeO_2_/β″‐Al_2_O_3_ 7:3 electrolyte‐based SIFC at 350–550 °C and the e) CeO_2_ electrolyte at 450–550 °C. f) Arrhenius plots for the CeO_2_/β″‐Al_2_O_3_ 7:3 and the pure CeO_2_ electrolyte. Impedance spectra of the g) CeO_2_/β″‐Al_2_O_3_ 7:3 fuel cells in hydrogen/air at 350–550 °C and h) CeO_2_ fuel cells at 450–500 °C, and their corresponding equivalent circuit models for fitting. i) Ro, Rp for CeO_2_/β″‐Al_2_O_3_ 7:3 and CeO_2_ SIFCs at 450, 500, and 550 °C.

To demonstrate the excellent electrochemical performance of CeO_2_/β″‐Al_2_O_3_ composite materials, current–voltage (*I–V*) and current–power (*I–P*) curves and electrochemical impedance spectroscopy (EIS) were applied to fuel cells with CeO_2_, β″‐Al_2_O_3_, and CeO_2_/β″‐Al_2_O_3_ composites as electrolytes. The results of the SIFC device are shown in **Figure** **2**. The designed CeO_2_/β″‐Al_2_O_3_ heterostructure exhibited higher performance at both 550 and 350 °C (Figure 2a). In Figure S4 (Supporting Information), *I–V* and *I–P* curves of the CeO_2_/β″‐Al_2_O_3_ heterostructure SIFC were recorded under different donor and acceptor material ratios, and the heterostructure SIFC had the highest power output of up to 1009 mW cm^−2^ (550 °C) at a metal ion molar ratio (Ce^4+^:Al^3+^) of 7:3. In addition, Figure 2b shows the good long‐term stability of CeO_2_/β″‐Al_2_O_3_ 7:3 cell devices at 550 °C. At a stationary current density of 156.875 mA cm^−2^, the voltage was maintained at ≈0.8 V for ≈50 h. However, as shown in Figure 2c, pure CeO_2_ fuel cells were operated at a current density of 156.875 mA cm^−2^, and the voltage sharply decreased after only maintaining it for 2 h. This occurred because the CeO_2_ electrolyte always had short‐circuit problems due to the reduction of CeO_2_. When we introduced the insulator β″‐Al_2_O_3_ to compose the heterostructure, the LEF in the constructed heterostructure could improve the ionic transportation to maintain fuel cell output and partially solve the instability problem by suppressing electronic conductivity. Therefore, in contrast to a pure CeO_2_ electrolyte devoid of heterointerface, employing β″‐Al_2_O_3_ to construct a heterostructure substantially improves the conductivity of oxygen ions. The LEF and C/H at the heterointerface contribute to prolonged conductivity duration for conducting oxygen ions.

The optimum ratio (CeO_2_/β″‐Al_2_O_3_ 7:3) and pure CeO_2_ electrolyte fuel cells were further investigated at various temperatures, and their *I–V*/*I–P* characteristics are shown in Figure 2d,e, respectively. For CeO_2_/β″‐Al_2_O_3_ SIFC, an OCV of 1.144 V and a remarkable maximum power density (MPD) of 1009 mW cm^−2^ were obtained at 550 °C. At the same time, this fuel cell achieved low‐temperature operating capability with power outputs of 387, 212, and 85.9 mW cm^−2^ at 400, 370, and 350 °C, respectively. However, as shown in Figure 2e, the corresponding MPD of CeO_2_‐based SIFC at 550 °C was 776.5 mW cm^−2^, and the *I–V*/*I–P* curve could not be measured at temperatures below 450 °C. To visually showcase the superior output performance of SIFC devices synthesized with CeO_2_/β″‐Al_2_O_3_ electrolyte, Table S2 (Supporting Information) compares the electrochemical output performance of various outstanding SIFC designs developed over the past two years. Evidently, our battery not only delivers higher power density at 550 °C but also offers additional advantages at 400 and 350 °C. In addition, Figure 2f shows the temperature dependence of the ion conductivity plots for the BP 7:3 and CeO_2_ electrolytes. Because the ionic resistance could be estimated based on the slope of the polarization curves (Figure 2d,e) in the ohmic polarization region,**^[^** ^43^ **^,^** ^44^ **^]^** pure CeO_2_ exhibited an ionic conductivity of 0.07–0.163 S cm^−1^ at 450–550 °C, and after compositing with β″‐Al_2_O_3_, the improvement values of BP 7:3 were 0.019–0.197 S cm^−1^ at 350–550 °C. The ionic conductivity reflected the ion flow resistance in the electrolyte, thus indicating that the CeO_2_/β″‐Al_2_O_3_ composite material had the ability to quickly transport ions as well as excellent high performance at low temperatures.

To better identify the originality of the fuel cell performance, the electrochemical impedance spectra (EIS) of the CeO_2_/β″‐Al_2_O_3_ 7:3 and CeO_2_ fuel cells were measured under open circuit voltage conditions in a H_2_/air atmosphere. Typical EIS spectra of the fuel cells measured at 350–550 °C are presented in Figure 2g,h, and the equivalent circuit of R_0_(R_1_Q_1_)(R_2_Q_2_) was used to fit the experimental data. In the EIS pattern, the obtained curves included three dominant processes, where the intersection at high frequencies was related to the ohmic resistance (R_0_). The semicircles or arcs at the mid‐ and low‐frequency regions corresponded to the charge transfer resistance (R_1_) and mass transfer resistance (R_2_), respectively. The sum of R_1_ and R_2_ represented the polarization resistance (R_P_). According to the fitting results summarized in Table S3 (Supporting Information), R_0_ and R_p_ at 550–450 °C are compared in Figure 2i; the R_0_ (0.150, 0.167, and 0.202 Ω cm^−2^) of the fuel cell using the CeO_2_/β″‐Al_2_O_3_ composite electrolyte was much lower than that of the CeO_2_ fuel cell (0.237, 0.275, and 0.347 Ω cm^−2^), respectively. This result indicated that the heterostructure composite electrolyte had a lower total bulk resistance, which was attributed to the formation of the heterointerface**^[^** ^37^ **^]^** and the accelerating high ionic conduction caused by the LEF and the melting C/H at the heterointerface between CeO_2_ and β″‐Al_2_O_3_.

Moreover, when the electrodes are NCAL with high electrode reactivity and the carbonate/hydroxide composite is in a molten state within this temperature range (550–450 °C), the R_p_ of the CeO_2_/β″‐Al_2_O_3_ fuel cell (0.236, 0.275, and 0.347 Ω cm^−2^) is also lower than that of the CeO_2_ fuel cell (0.240, 0.381, and 0.501 Ω cm^−2^). The lower R_p_ can be attributed to enhanced grain boundary conductance and rapid electrode reactions in the triple phase boundary (TPB) regions.**^[^** ^22^ **^,^** ^43^ **^]^** However, at 350 °C, as shown in Table S3a (Supporting Information), the R_p_ in CeO_2_/β″‐Al_2_O_3_ fuel cells suddenly increases by 31.57 Ω cm^−2^. This may be because carbonates/hydroxides gradually transform into a condensed state at this time. Figure S5 (Supporting Information) shows the surface and cross‐sectional SEM images of the surface morphology of the CeO_2_/β″‐Al_2_O_3_ samples before and after testing. The AP sample tested at room temperature appears denser due to the coverage of C/Hs, which hinders the transmission path of oxygen from the electrode to the TPB. This greatly increases the mass transfer resistance of oxygen (R_2_); thus, the rapid increase in R_2_ leads to an increase in R_p_, which in turn damages the oxygen reduction reaction (ORR) of the electrode and deteriorates the performance of the SIFC.**^[^** ^45^ **^]^**

### 2.4 FT–IR, TG‐DSC and O_2_‐TPD/MS Analysis

**Figure 3:**
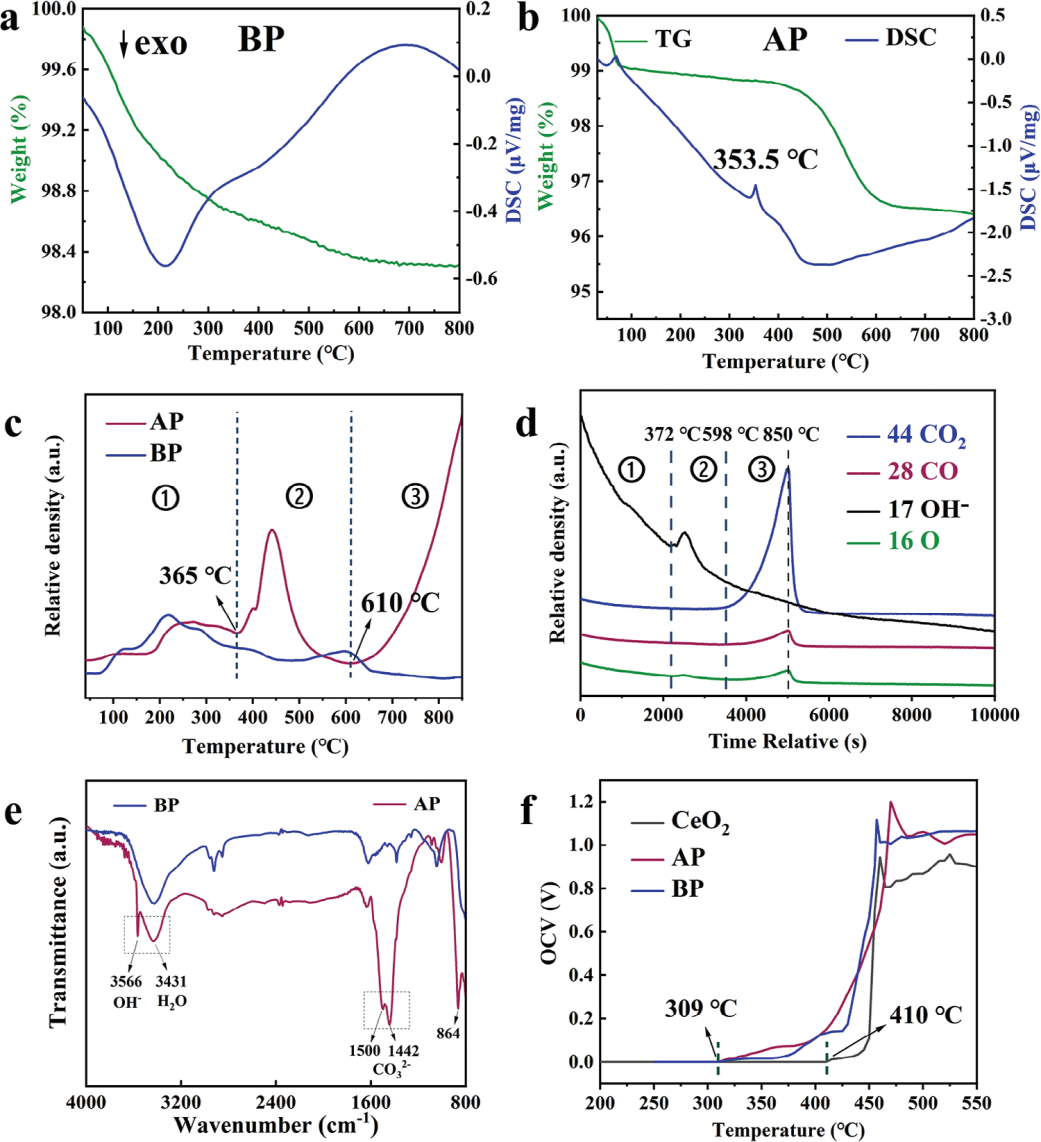
Analysis of the manipulating mechanism of oxygen ion conduction for SIFC at 350 °C. Differential scanning calorimetry and thermogravimetric analysis (TG‐DSC) curves of the a) BP and b) AP powders, c) O_2_ temperature‐programmed desorption (O_2_‐TPD) profiles of the BP and AP powder and d) mass spectra (MS) signals of AP powder. e) Fourier transform infrared spectroscopy (FT‐IR) spectrogram of the BP and AP powders. f) OCV temperature dependence curves of the fuel cells with AP, BP, and CeO_2_ samples as electrolytes in the temperature range of 200–550 °C.

To further investigate the reason why the C/H is dedicated to optimizing interfacial oxygen ion conduction in CeO_2_/β″‐Al_2_O_3_ heterostructure electrolyte fuel cell at ≈350 °C, a comparative assessment of differential scanning calorimetry and thermogravimetric analysis (TG–DSC) is applied to examine the thermal stability of the electrolyte powder components. **Figure** **3**a shows the TG‐DSC curves of the BP sample, where the weight loss is attributed to the net loss of oxygen with increasing temperature.**^[^** ^39^ **^]^** As shown in Figure 3b, a good correspondence exists between the endothermic peak at 353.5 °C on the DSC curve and the weight loss step on the TG curve, which is caused by the melting and final decomposition of a certain material. Moreover, to determine the molten substance, the O_2_‐TPD‐MS study is performed on the BP and AP samples. In Figure 3c, the O_2_‐TPD curve for AP exhibits a desorption peak in the second stage (365–602 °C), while Figure 3d shows the MS signal of O_2_‐TPD for AP. Clearly, at 365–602 °C, a peak with exactly the same shape appears in the curve with a molecular weight of 17 (─OH). In the third stage (602–850 °C), the high intensity peaks in the O_2_‐TPD can be interpreted by the MS signals with molecular weights of 44 (CO_2_) and 28 (CO). Combined with the XPS analysis, the O_2_‐TPD‐MS results indicate that the melted and decomposed substances are hydroxides in stage 2 and carbonates in stage 3, respectively. In 2021, Chen et al. reported that the melting/solidification critical point of a LiOH/Li_2_CO_3_ mixture was ≈419 °C.**^[^** ^21^ **^]^** However, in the CeO_2_/β″‐Al_2_O_3_ heterostructure electrolyte, the melting point of the hydroxide/carbonate mixture decreases to 353.5 °C. In Figure S6 (Supporting Information) shows the TG‐DSC analysis results of the multicomponent mixture. Additionally, in the fuel cell testing environment, when the NCAL anode and the β″‐Al_2_O_3_ located at the three‐phase boundary on one side of the anode come into contact with moist hydrogen gas, they undergo a reduction reaction. The alkali metal (Li, Na, and Mg) thus further reduced reacts with water to form molten hydroxides of elements like Li, Na, and Mg.**^[^** ^40^ **^]^** These liquids subsequently permeate into the electrolyte via capillary diffusion. During this process, they interact with CO_2_, leading to the formation of carbonates and resulting in complexes of hydroxides of Li, Na, and Mg with carbonates (C/H). Experimental evidence has shown that the melting temperature of these complexes occurs at 353.5 °C. This is significantly lower than the melting point temperature of the Li_2_CO_3_/LiOH complex in traditional SIFCs (419 °C). Finally, a mixed state consisting of metal (Li, Na, and Mg) hydroxides and carbonates is formed, and its final melting point is 353.5 °C. To further identify the chemical states and multiple components, Figure 3e shows the Fourier transform infrared spectroscopy (FT–IR) spectrogram of the BP and AP powders. The absorption peaks at 3566 and 3431 cm^−1^ belong to the hydroxyl stretching vibrations from hydroxide and H_2_O,**^[^** ^21^ **^,^** ^41^ **^]^** the strong peaks located at 1500 and 1442 cm^−1^ are attributed to the C─O antisymmetric stretching vibration, and the peaks at 864 cm^−1^ correspond to the out‐of‐plane flexural vibration of CO_3_^2−^.**^[^** ^19^ **^,^** ^36^ **^,^** ^41^ **^]^** Therefore, the FT–IR result demonstrates the presence of hydroxides and carbonates of elements, such as Li, Na, and Mg in the AP powders. The resulting composite has been shown to melt and form a eutectic liquid at temperatures above 419 °C, ultimately penetrating into the electrolyte interior to form a multicomponent AP powder.**^[^** ^21^ **^]^** Moreover, Figure 3f shows the temperature dependence curves of the open circuit voltage (OCV) during the heating process of fuel cells using the BP, AP, and CeO_2_ electrolytes in the range of 200–550 °C. The OCV inflection point positions of the composite electrolyte cells are ≈100 °C lower than that of the pure CeO_2_ electrolyte fuel cell. For the CeO_2_ electrolyte fuel cells, the solidification of carbonates/hydroxides in the electrolyte results in the absence of OCV with temperatures below 410 °C, thus, the fuel cell cannot operate at this temperature. In the CeO_2_/β″‐Al_2_O_3_ electrolyte fuel cell, the melting point of carbonates/hydroxides decreases to 353.5 °C, and OCV occurs with temperatures above 309 °C; this results in a high MPD of 85.7 mW cm^−2^ in the composite electrolyte fuel cells at 350 °C. Therefore, the ionic conductivity of the CeO_2_/β″‐Al_2_O_3_ electrolyte significantly improved at 350–450 °C, and the minimum working temperature decreased from 450 to 350 °C. However, in Figure S3 (Supporting Information), when pure β″‐Al_2_O_3_ was used as the electrolyte, molten complexes were present inside the electrolyte bulk. The fuel cell device only exhibited 186 mW cm^−2^ at 550 °C and showed almost no power at 450 °C. Therefore, this result indicated that the enhancement effect of molten hydroxides/carbonates was a passive response rather than an active behavior. In 2020, A. Mondal et al. molecular dynamics simulations with a developed force field to determine the ionic conductivity of molten alkali metal carbonate‐hydroxide mixtures across various cation and hydroxide compositions. The findings suggest that increasing the quantity of hydroxides decreases viscosity while increasing ionic conductivity.**^[^** ^54^ **^]^** At this elevated temperature, C/H in its melted form facilitates LEF's standard function, enabling accelerated transport of oxygen ions within the fuel cell composite electrolyte. Meanwhile, as a gate for high‐speed oxygen ion conduction in a continuous phase interface network, the network in a molten state ensures the transmission of the conductive particles at the interface.**^[^** ^21^ **^,^** ^25^ **^,^** ^36^ **^,^** ^40^ **^,^** ^42^ **^]^**

### 2.5 Band Energetics and Heterostructure Analysis

Compared with pure CeO_2_ fuel cells, the electrochemical performances of CeO_2_/β″‐Al_2_O_3_ heterostructure electrolyte fuel cells were significantly improved in terms of OCVs and MPDs (Figure 2). Its mechanism of action was shown to be a bandgap alignment in heterostructure composite electrolytes. At the heterointerface of two‐phase materials, LEF helped prevent electron migration while enhancing ionic conduction. To verify the assumptions, the UV–vis absorption spectra and UPS cutoff images of CeO_2_ and β″‐Al_2_O_3_ were used for bandgaps and valence band maxima (VBM) testing.

**Figure 4:**
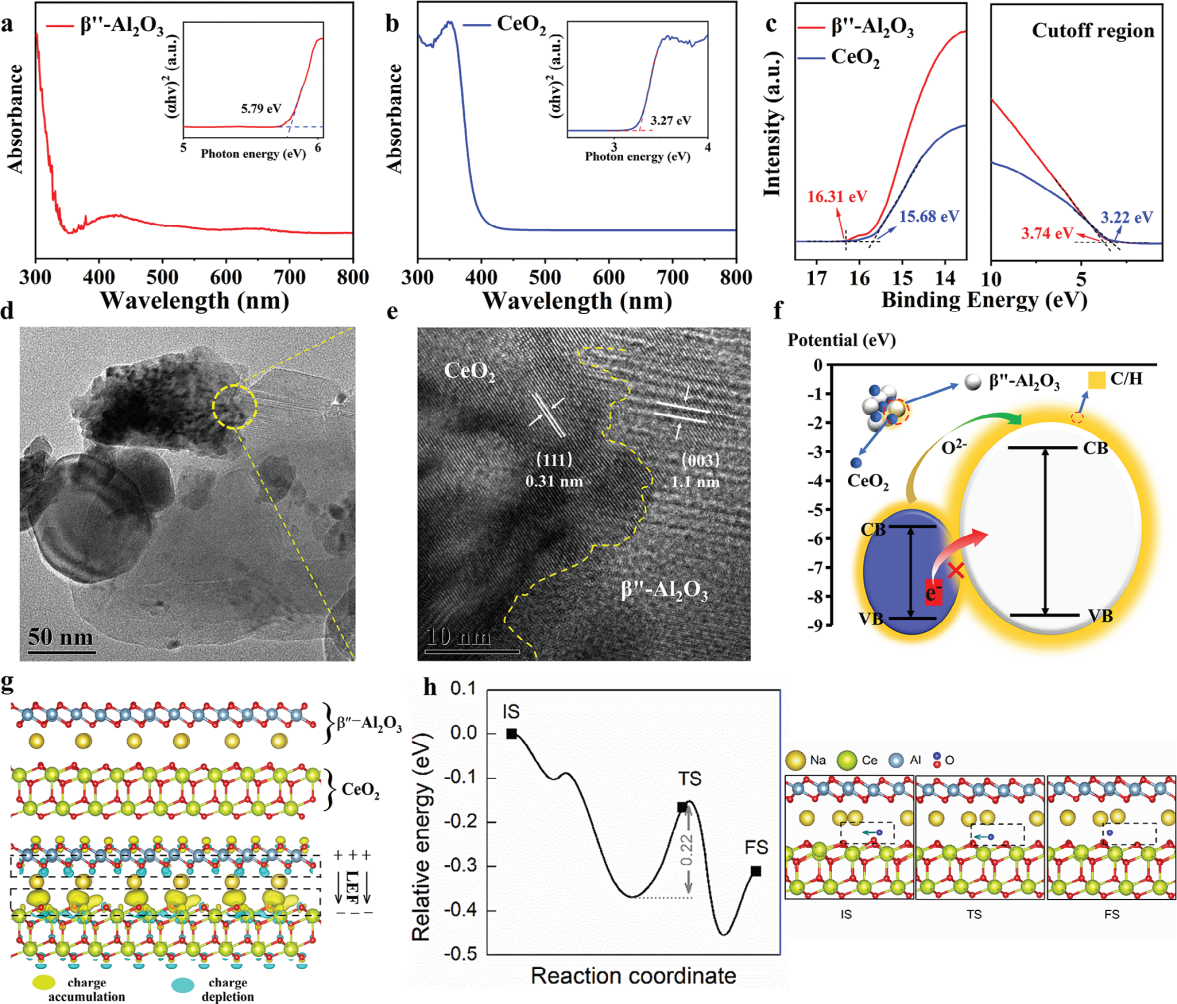
Fast oxygen ion conduction at the heterointerface of CeO_2_/β″‐Al_2_O_3_. UV–vis absorption spectra and calculated bandgaps for CeO_2_ a) and β″‐Al_2_O_3_ b). c) UPS plots of CeO_2_ and β″‐Al_2_O_3_ with magnified views of the low‐binding energy cutoff and the high‐binding energy cutoff regions. d) TEM and e) HR‐TEM images of the BP powder. f) Schematic diagram of the potential energy barrier in the CeO_2_/β″‐Al_2_O_3_ electrolyte solid oxide fuel cell. g) Charge density differences at the CeO_2_/β″‐Al_2_O_3_ interface. h) Relative energy diagram for oxygen diffusion at the CeO_2_/β″‐Al_2_O_3_ interface. The optimized atomic geometries of the initial state (IS), transition state (TS), and final state (FS) along the reaction coordinate are shown in the right panel.

**Figure** **4**a,b show the UV–vis absorption spectra of CeO_2_ and β″‐Al_2_O_3_ according to the following function:

(1) $$\alpha h\nu =A\;{\left(h\nu -E_{g}\right)}^{n}$$

where α is the absorption coefficient, *h*ν is the energy of photons, A is a constant, n is 1/2 for a direct bandgap semiconductor and *E*_g_ is the bandgap energy. The obtained band gap values in the inset of Figure 4a,b show that the bandgaps for CeO_2_ and β″‐Al_2_O_3_ are 3.27 and 5.79 eV, respectively. Moreover, the VBM can be obtained according to the following equation:

(2) $$VB=21.2\;\mathrm{eV}-\left(SC-V_{VBE}\right)$$

where 21.2 eV is the incident He source energy, *V_VBE_* represents the energy difference between the Fermi level and VBM edge, and SC represents a secondary cutoff edge. According to the results shown in Figure 4c, the VBM levels of CeO_2_ and β″‐Al_2_O_3_ are −8.74 and −8.63 eV, respectively. Thus, the corresponding conduction band (CB) bottom levels are determined to be −5.47 and −2.84 eV. Based on these results and our previous work**^[^** ^37^ **^]^** and Afanas'ev V's report,**^[^** ^46^ **^]^** electron barriers can form at interfaces between various high‐mobility semiconductors and insulating metal oxides. Moreover, Figure 4d shows the typical heterointerface between the two materials, along with the portion marked with a magnified yellow circle; a zoomed image of this circle is shown in Figure 4e, and lattice stripes of CeO_2_ and β″‐Al_2_O_3_ are clearly observed. The calculated main exposed lattice spacings are ≈0.31 and 1.1 nm, corresponding to the (111) plane of CeO_2_ and the (003) plane of β″‐Al_2_O_3_, respectively.**^[^** ^29^ **^,^** ^47^ **^]^** Therefore, when the microscale heterointerfaces are widely formed between two material phases, the LEFs will be automatically constructed, as shown in Figure 4f. This potential energy barrier will help block the electronic transmission of the SOFC bulk, which will significantly suppress the problem of fuel cell short circuits and achieve high OCVs of fuel cells (from 1.04 V CeO_2_ to 1.144 V CeO_2_/β″‐Al_2_O_3_ fuel cells at 550 °C).

**Figure 5:**
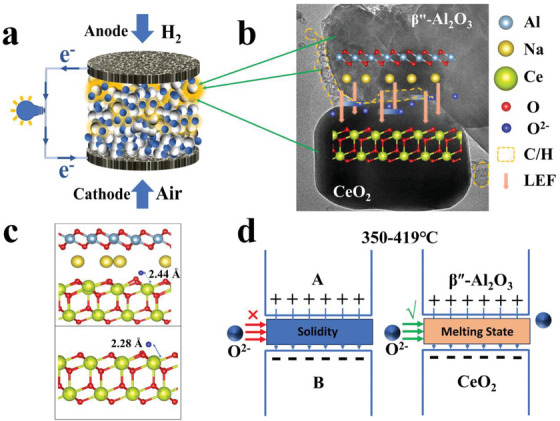
Design of the CeO_2_/β″‐Al_2_O_3_ heterostructure composite electrolyte with the synergistic effect of the LEF and C/H composites. a) Schematic diagram of the CeO_2_/β″‐Al_2_O_3_ electrolyte solid oxide fuel cell. The white particles are β″‐Al_2_O_3_, the blue particles are CeO_2_, and the yellow emitting parts are C/H composites. b) Schematic of the CeO_2_/β″‐Al_2_O_3_ fuel cell and its operation mechanism; a LEF built at the CeO_2_/β″‐Al_2_O_3_ interface from the β″‐Al_2_O_3_ side to the CeO_2_ side acts on accelerated oxygen ion transport, and the C/H composites serve as a gate to control the transport of oxygen ions at heterogeneous interfaces. c) Adsorption distance for the oxygen ion on the interface of the CeO_2_/β″‐Al_2_O_3_ heterostructure of 2.44 Å, and the CeO_2_ lattice with the (111) plane is 2.28 Å. d) The oxygen ionic migration principle in heterointerface of the typical composite electrolyte (left) and CeO_2_/β″‐Al_2_O_3_ electrolyte (right).

DFT calculations are performed to further understand the manipulating interfacial oxygen ion conduction mechanism. Figure 4g shows the charge density difference map for the formation of the CeO_2_/β″‐Al_2_O_3_ heterostructure. An unbalanced charge distribution is present between the CeO_2_ and β″‐Al_2_O_3_ layers, and electrons accumulate on the CeO_2_ side, which provides the strong charge transfer between CeO_2_ and β″‐Al_2_O_3_. Then, the unbalanced charge distribution renders a local electric field (LEF) within the CeO_2_/β″‐Al_2_O_3_ interface region; additionally, the LEF constructed at the heterogeneous interface of the two‐phase material effectively regulates the transport mode of oxygen ions, weakens the binding strength of oxygen ions, and accelerates oxygen ion transport. **Figure** **5**c shows that the adsorption distance for the oxygen ion on the CeO_2_ side of the interface is 0.16 Å longer than that on the clean CeO_2_ (111) surface, corroborating that the CeO_2_/β″‐Al_2_O_3_ heterostructure can provide a weak bonding interaction for the ions and thus build a highway for ion transport. This deduction is understandable because a longer adsorption distance can reduce the energy barrier for oxygen ion transfer at the interface. As shown in Figure 4h, the energy barrier for oxygen ion diffusion from the Ce site in the CeO_2_/β″‐Al_2_O_3_ interface to the adjacent Ce site is 0.22 eV, indicating that diffusion is thermodynamically favorable at the working temperatures. Consequently, these results are consistent with the experimental observations of the lower ohmic resistance and high MPD of the CeO_2_/β″‐Al_2_O_3_ heterostructure composite electrolyte SIFC. Therefore, combined with the enhanced ionic conductivity and the significant improvement of low‐temperature properties, our results achieved the fast oxygen ion conduction at the heterointerface of CeO_2_/β″‐Al_2_O_3_.

Concluding this article, Figure 5 presents a comprehensive schematic structure of the SIFC's operation mechanism incorporating the CeO_2_/β″‐Al_2_O_3_ heterostructure composite electrolyte. In Figure 5a, the LEF constructed at the heterogeneous interface of the two‐phase material effectively regulated the mode of oxygen ion transport and acted on the accelerated oxygen ion transport (Figure 5b). It's important to mention that the electrolyte in SIFC is pressed using the dry pressing method and has not undergone sintering, leaving numerous voids within. As a result, during the electrochemical testing of the fuel cell, molten C/H permeates due to concentration gradients, ultimately establishing an ion‐conductive network with alternating distributions of heterogeneous interfaces and C/H. The capillary permeation of C/H happens haphazardly, leading to an uneven distribution of C/H at these interfaces. Where the two materials closely contact, the LEF expedites oxygen ion transport across the interface. If there's a rupture in the interface between the materials, molten C/H continues to facilitate oxygen ion transfer. This ensures the fuel cell's functionality until the C/H solidifies (>353.5 °C). From Figure 5c, the adsorption distance for the oxygen ion on the CeO_2_ side of the interface was 0.16 Å longer than that on the clean CeO_2_ (111) surface, corroborating that the CeO_2_/β″‐Al_2_O_3_ heterostructure could provide a weak bonding interaction for the ions and thus build a highway for oxygen ion transport. Simultaneously, illustrated in Figure 5d, the C/H within the heterointerface of a typical heterostructure composite electrolyte is characterized by solidity (melting point at 419 °C), this significantly impairs the migration behavior of oxygen ions, severely diminishing the conductivity and stability of the SIFCs. However, our research mentioned above indicates that the melted composites of C/H are needed to ensure the enhanced ion transport caused by LEF at the interface of the heterostructure. Hence, in the as designed CeO_2_/β″‐Al_2_O_3_ heterostructure electrolyte, due to the melting point of the C/H at the interface occurring at 353.5 °C, this temperature ensures stable ion transfer of SIFCs at 350–550 °C. Therefore, the developed CeO_2_/β″‐Al_2_O_3_ electrolyte exhibited higher fuel cell performance than pure CeO_2_ electrolyte fuel cells at 450–550 °C and showed outstanding electrochemical performance in the lower temperature range (350–450 °C). Our CeO_2_/β″‐Al_2_O_3_ heterostructure design effectively regulates the heterointerface oxygen ion conduction of composite electrolytes at low temperatures and guides interface engineering to achieve the optimal electrochemical performances at temperatures of 350–550 °C for SIFCs.

## 3 Conclusion

In conclusion, we've developed a novel CeO_2_/β″‐Al_2_O_3_ electrolyte exhibiting superior low‐temperature oxygen ion conductivity due to construction of LEF and modulation of the C/H complex melting point. The resulting SIFC device achieves a peak power density of 1W cm^−2^ at 550 °C with remarkable durability of almost 50 h. Different from conventional SIFC losing functionality at 350 °C, this CeO_2_/β″‐Al_2_O_3_ design boasts an impressive ionic conductivity of 0.019 S cm^−1^ and power output of 85.9 mW cm^−2^. Experimental results and DFT calculations suggest that the superior electrochemical performance is due to the LEF at the CeO_2_/β″‐Al_2_O_3_ heterointerfaces, promoting efficient ionic transport while suppressing electronic conduction. Meanwhile, the molting point of C/H complexes of alkali metal elements (Li, Na, and Mg) is reduced to 353.5 °C, which functioning as a rapid ion transport valve and significantly enhances the electrochemical performance of SIFC at lower temperatures. This research has successfully demonstrated rapid oxygen ion transport at low temperatures, providing a practical approach for creating high‐performance, low‐temperature SIFC electrolytes.

## Conflict of Interest

The authors declare no conflict of interest.

## Author Contributions

Y.Z. and D.Z. contributed equally to this work. Y.Z., D.Z., and C.Z. conceived the research concept. Y.Z. and D.Z. conducted the experiments. Z. Z. conducted DFT calculations. C.Z. and W.L. directed the overall project. Y.Z. and D.Z. contributed to the characterization. J.L., Y.O., J.Y., Z.L., X.B., and N.W. analyzed the data. Y.Z., D.Z., L.Z., W.L., and C.Z. wrote the manuscript. All the authors have read and commented on the manuscript.

## Supporting information

Supporting Information

## Data Availability

The data that support the findings of this study are available from the corresponding author upon reasonable request.
