# Supplementary material for: Observation of Fast Low‐Temperature Oxygen Ion Conduction in CeO2/β"‐Al2O3 Heterostructure
Source: Adv Sci (Weinh). 2024 Jul 21;11(35):2401130. doi: 10.1002/advs.202401130 (PMC11425223; doi:10.1002/advs.202401130)
Supplement: Supplementary file 1 — Supporting Information [file ADVS-11-2401130-s001.docx]

**Supplementary Information**

Observation of Fast Low-temperature Oxygen Ion Conduction in CeO_2_/β"-Al_2_O_3_ Heterostructure

Yingbo Zhang‡^1^, Decai Zhu‡^1^, Zhonglong Zhao^1^, Jiamei Liu^1^, Yuzhao Ouyang^1^, Jiangyu Yu^1^, Zhongqing Liu^1^, Xixi Bai^1^, Nan Wang^1^, Lin Zhuang*^3^, Wuming Liu^2^, Chengjun Zhu*^1^

^1^Key Laboratory of Semiconductor Photovoltaic Technology and Energy Materials of Inner Mongolia Autonomous Region, School of Physical Science and Technology, Inner Mongolia University, 235 West Daxue Street, Hohhot, Inner Mongolia, 010021, People’s Republic of China.

^2^Beijing National Laboratory for Condensed Matter Physics, Institute of Physics, Chinese Academy of Sciences, Beijing, 100190, People’s Republic of China.

^3^State Key Laboratory of Optoelectronic Materials and Technologies, School of Physics, Sun Yat-Sen University, Guangzhou 510275, People’s Republic of China.

Corresponding author, *Email: [cjzhu@imu.edu.cn](mailto:cjzhu@imu.edu.cn) (C. Zhu). [stszhl@mail.sysu.edu.cn](mailto:stszhl@mail.sysu.edu.cn) (L. Zhuang)

‡Yingbo Zhang and Decai Zhu contributed equally to this work.

**CONTENTS**

**Experimental Procedures 3-7**

**S1.** **XRD patterns of β"-Al_2_O_3_ at the different sintering temperature and AP powder. 8**

**S2. The energy-dispersive X-ray spectroscopic (EDS) mapping of the main elements of the heterostructure composite 9**

**S3. IV-IP curves of fuel cells of pure β"-Al_2_O_3_ electrolyte 10**

**S4. IV-IP curves of fuel cells based on composite electrolytes of different proportions 11**

**Table S2. Comparison of fuel cell performance between this work and advanced SIFC in the literature. 12**

**S5. Surface and cross-sectional SEM images of the surface morphology of BP and AP samples 13**

**S6. TG-DSC curves of various combinations of powders 14**

**Table S3. The impedance fitting results of BP 7:3 and pure CeO_2_ fuel cell 15**

**Supplementary references 16**

**Experimental Procedures**

**Materials synthesis**

Na_1.67_Mg_0.67_Al_10.33_O_17_ (β"-Al_2_O_3_) was synthesized by the sol-gel method with NaNO_3_, Mg(NO_3_)_2_, and Al(NO_3_)_3_·9H_2_O as the raw materials. Appropriate amounts of several analytical reagents were dissolved in deionized water to make solution 'a,' PVP-10000 was weighed at twice the molar content of all cations and dissolved in deionized water as solution 'b,' and an equal molar content of ethylene glycol was used as a stabilizer. 'a' and ethylene glycol was slowly added dropwise into transparent 'b' to obtain a mixed solution, and the pH value and metal ion concentration of the mixed solution was adjusted to 3 and 0.2 mol/L, respectively. The resulting solution was stirred at 80°C for 4 hours to produce a reddish-brown viscous gel. This gel was dried at 120°C for 12 hours, resulting in a precursor powder. The precursor powder was then gradually heated to 500°C at a rate of 1°C/min and held at that temperature for 2 hours. Finally, the powder was calcined at 1400°C for 2 hours to obtain the β"-Al_2_O_3_ powders. The procedure was the same as in previously reported experiments **^[1]^**, and the experimental setup was consistent with the literature.

CeO_2_ was synthesized by a carbonate coprecipitation method with Na_2_CO_3_ as the precipitant. In the synthesis process, a solution of 0.15 mol Ce(NO_3_)_3_•6H_2_O in 300 mL of deionized water and a solution of 0.2 mol Na_2_CO_3_ in 200 mL of deionized water were separately prepared. Then, the nitrate solution was slowly added dropwise to the sodium carbonate solution under mild stirring to form carbonate precipitants. The obtained white sediment was aged for 10 hours, filtered and washed three times with deionized water. Then, the precipitate was dried at 120℃ for 24 hours and calcined in air at 800℃ for 2 hours to obtain the CeO_2_ powders.

To prepare the composite electrolyte powder, the prepared CeO_2_ and β"-Al_2_O_3_ were first dissolved in ethanol. After sonication for 30 minutes, the resulting solution was ball milled for 4 hours. Then, the mixture was dried at 120°C, and the powder was sintered at 550°C for 5 hours. Finally, the powder was thoroughly ground to obtain a uniform composite electrolyte powder. Samples with varying molar ratios of Ce^4+^ and Al^3+^ ions, namely, 9:1, 8:2, 7:3, and 6:4, are denoted by the nomenclature 'CeO_2_/β"-Al_2_O_3_ x:y'. The raw material powder with a 7:3 ratio is designated as 'BP', while the electrolyte obtained from the fuel cell after electrochemical performance is marked as 'AP'.

Table S1. Mass ratio of pure CeO_2_ and CeO_2_/β"-Al_2_O_3_ composites in different metal ions mole ratios.

| **Composite** | Pure CeO_2_ | 9:1 | 8:2 | 7:3 | 6:4 | 5:5 |
| --- | --- | --- | --- | --- | --- | --- |
| mass ratio(g) |  | 26:1 | 12:1 | 7:1 | 4:1 | 3:1 |

**Fuel cell fabrication and test**

The fuel cell devices were fabricated using the dry-pressing method. First, 10 g LiNi_0.8_Co_0.15_Al_0.05_O_2_ (NCAL) (Tianjin Bamo) and 3 mL terpineol were uniformly ground to form a slurry; the slurry was coated on the nickel foam and then desiccated at 120°C for 40 min to obtain an electrode (Ni-NCAL). CeO_2_ or CeO_2_/β"-Al_2_O_3_ powder (0.30 g) was used as the electrolyte and was compacted between the two electrodes under a load of 400 MPa for 90 s. The obtained SOFC devices had a Ni-NCAL/electrolyte/NCAL-Ni sandwich structure, with a fuel cell diameter of 13 mm and an active area of 0.64 cm^2^. Afterward, all fuel cells were preheated at 550°C for 30 min, and then the electrochemical performance was measured by an electronic load (IT8511, ITECH Electrical Co. Ltd., China) with hydrogen for fuel gas (125 mL min^-1^) and air as the oxidant gas.

**Long term stability testing:**

The instrument model currently used for chemical stability testing in our laboratory is an electronic load (IT8511, ITECH Electrical Co. Ltd., China). The hydrogen flow rate to 110 mL/min, and the test temperature is 550 ℃. During the stability test, the electronic load will apply a constant current to the battery, and then record the stability data of the voltage value over time. Before testing begins, the minimum constant current that can be selected is 0.1 A. Divide 0.1 A by the effective area of 0.64 cm^2^ and multiply by 1000 to obtain a constant current density of 156.25 mA/cm^2^. After the testing process begins, the instrument will slightly increase this value, therefore in the test data we obtained, the constant current density is 156.875 mA/cm^2^

Electrochemical impedance spectroscopy (EIS) measurements were performed by an electrochemical workstation (Zahner, Zennium-E, Germany) with an AC amplitude of 10 mV and a frequency range of 0.1-10^6^ Hz; the obtained impedance plots were fitted by ZSIMPWIN software.

**Material Characterization**

The crystal structures of the samples were characterized by an X-ray diffractometer (XRD, MiniFlex600, Rigaku, Japan) with Cu K_α_ radiation (λ =1.54060 Å; tube voltage = 45 kV; tube current = 15 mA). The morphological characteristics of the materials were analyzed by field emission scanning electron microscopy (FESEM, SU8220, Hitachi, Japan), and the elemental composition was characterized by an energy dispersive spectrometer (EDS, X-Max^N^, Oxford Instruments, U.K.). The high-resolution transmission electron microscopy images were obtained by a Tecnai f20 200 kV field emission high resolution transmission electron microscope (HR-TEM, FEI, America). The X-ray photoelectron spectra (XPS) and UV photoelectron spectroscopy (UPS) data were acquired via an X-ray photoelectron spectrometer (ESCALAB Xi+, Thermo Fisher Scientific, U.K.). The band gap of the sample was determined by ultraviolet−visible (UV−vis) absorption spectroscopy (UV, LAMBDA750s, PerkinElmer). The oxidation states of the various elements in the electrolyte samples were characterized by Fourier transform infrared spectroscopy (FTIR, VERTEX70, Bruker, Germany). Furthermore, differential scanning calorimetry and thermogravimetric analysis (TG-DSC, NETESCH, STA449 F3, Germany) were used to investigate the thermostability of various materials. TG/DSC test employs a flow of 30 mL/min of N_2_ as the protective gas and 30 mL/min of pure air as the blowing gas. Both the sample and reference containers are aluminum oxide crucibles, with a heating rate of 10 °C/min. The O_2_-TPD-MS measurements were carried out on a PCA-1200 chemisorption analyzer (O_2_-TPD, Builder Electronic, China) and an MS-200 mass spectrometer (MS, Builder Electronic, China). The O_2_-TPD/MS test utilizes 40 ml/min of helium gas as the carrier gas. Following dehydration of the test material, it undergoes no oxygen absorption treatment. The sample is positioned within a glass U-shaped tube and heated at a rate of 10°C/min. The desorbed products of the carrier gas are directly linked to the mass spectrometer to determine the relative molecular weight of the desorbed substances.

**DFT calculations**

DFT calculations were performed using the Vienna ab initio Simulation Package (VASP) with projector-augmented wave (PAW) pseudopotentials.**^[2,3]^** The exchange-correlation interaction was described by using the Perdew-Burke-Ernzerhof (PBE) functional.**^[4]^** The plane-wave energy cutoff was set to 400 eV, and the Brillouin zone was sampled with a 3×3×1 k-point mesh according to the Monkhorst-Pack scheme.**^[5]^** The CeO_2_/β"-Al_2_O_3_ heterostructure was modeled by a supercell comprising a 3×3 in-plane supercell of CeO_2_ (111) and a 2×2 in-plane supercell of β"-Al_2_O_3_. An effective on-site Coulomb repulsion U = 5.5 eV was applied to the 4*f* orbitals of Ce atoms according to the scheme of Dudarev et al. **^[6]^** The migration barrier energy of oxygen was calculated by the climbing image nudged elastic band (CI-NEB) method.**^[7]^**

The CeO_2_/β"-Al_2_O_3_” heterostructure was constructed by connecting the in-plane 3×3 supercell of CeO_2_(111) and 2×2 supercell of β"-Al_2_O_3_(0001). Nine Na atoms and one O atom were added between the CeO_2_(111) and β"-Al_2_O_3_(0001) slabs to simulation the ion conduction process.

**S1.** **XRD patterns of β"-Al_2_O_3_ at the different sintering temperature and AP powder.**


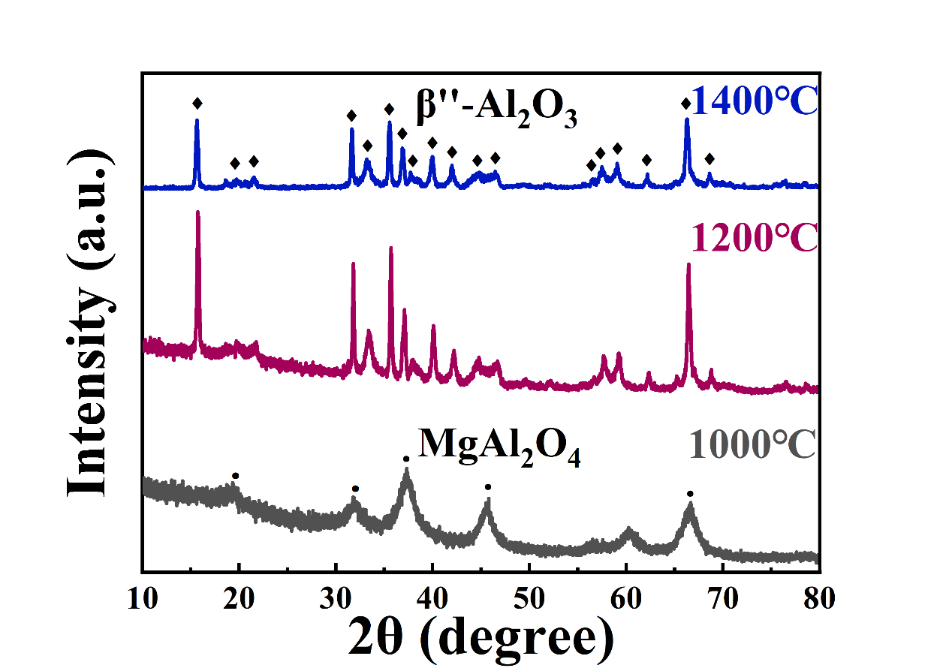


**Figure S1a.** XRD patterns of β"-Al_2_O_3_ powder at the temperature of 1000℃, 1200℃ and 1400℃, respectively. The crystalline phase of products at 1000°C is indexed to a magnesium aluminum oxide (PDF No.48-0528), and when the temperature up to 1200°C, diffraction of β"-Al_2_O_3_ appeared. Eventually, at 1400°C, the β"-Al_2_O_3_ with excellent crystallinity is obtained for synthesizing composite electrolyte materials.


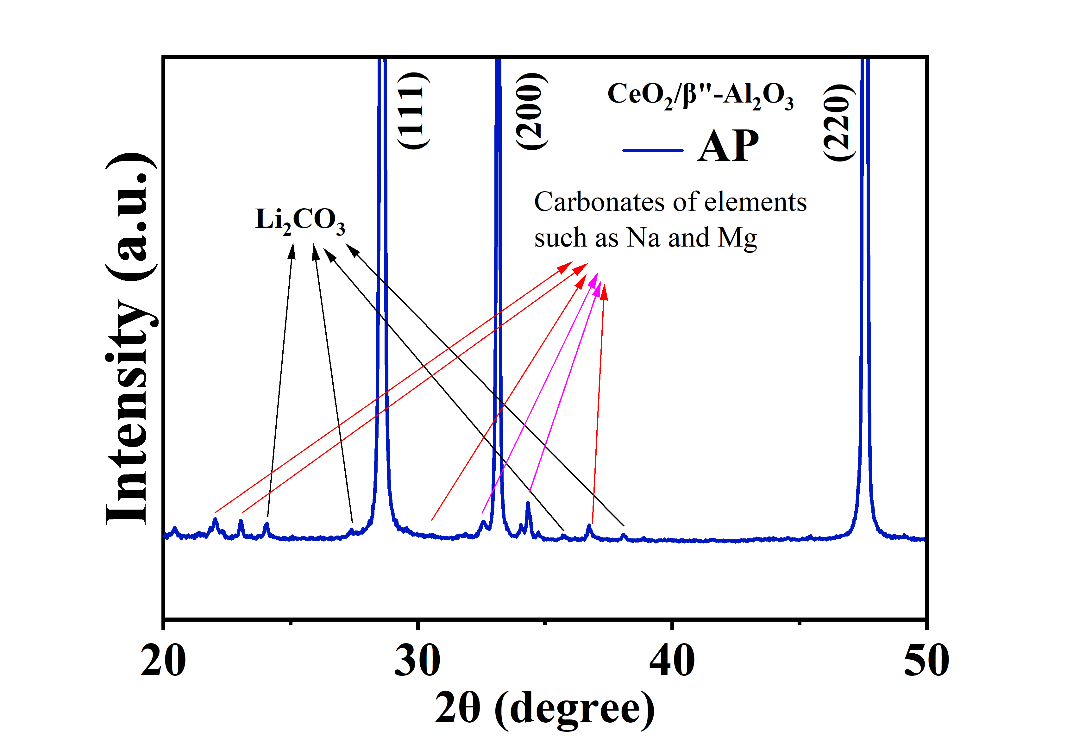


**Figure S1b.** XRD pattern of the AP (CeO_2_/β"-Al_2_O_3_ electrolyte after fuel cell test) powder.

**S2. The energy-dispersive X-ray spectroscopic (EDS) mapping of the main elements of the BP sample**


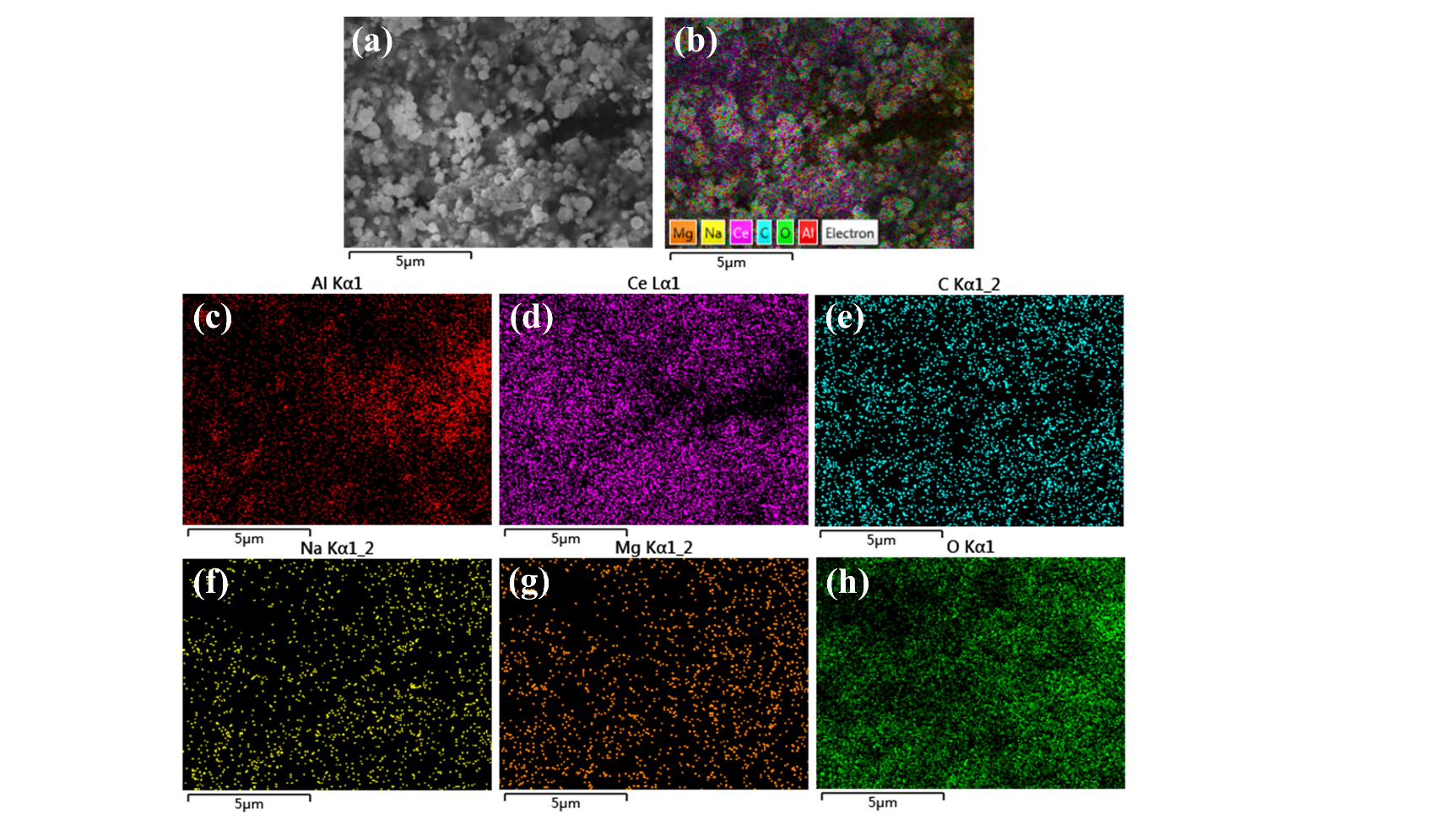


**Figure S2.** SEM image of BP powder (a) Overall element distribution b and individual distributions of (c) Al, (d) Ce, (e) C, (f) Na (g) Mg and (h) O, respectively.

As shown in the figure, Na, Mg, Al, Ce, and O are uniformly distributed throughout the entire region, and Ce and Al elements exhibit complementary positional distribution. This probes that the establishment of charge transport network between CeO_2_ and β"-Al_2_O_3_, which is beneficial for ion conduction.

**S3.** **IV-IP curves of fuel cells of pure β"-Al_2_O_3_ electrolyte**


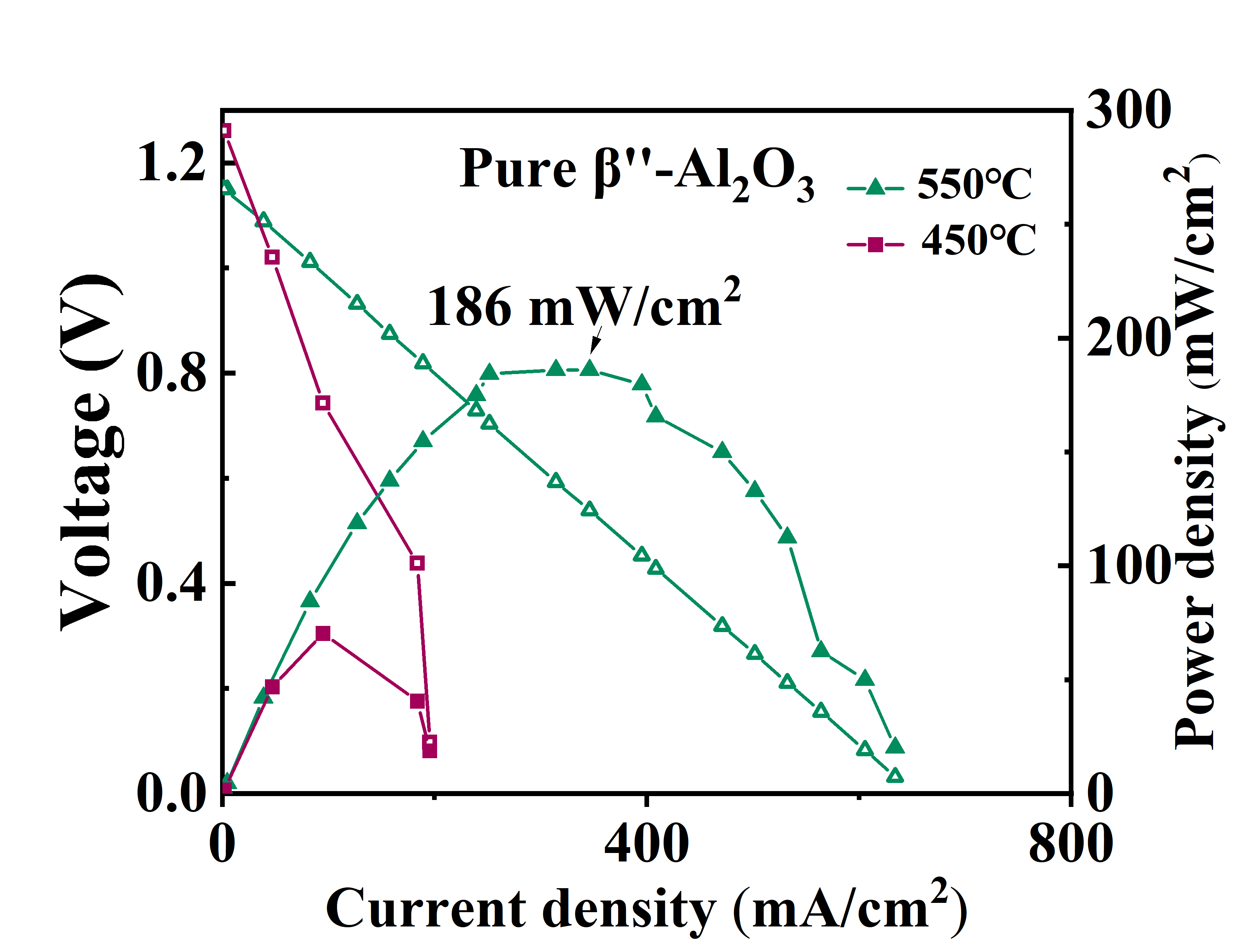


**Figure S3.** IV-IP curves of pure β"-Al_2_O_3_ at 550 and 450 ℃.

Sodium-β-alumina ceramics have been widely studied due to their sodium conductivity at the applications of the materials include electrolyte and sodium-ion separator in devices **^[1]^**. However, the fuel cell device only exhibits 186 mW/cm^2^ at 550℃ and almost no power at 450℃.

**S4. IV-IP curves of fuel cells based on composite electrolytes of different proportions**


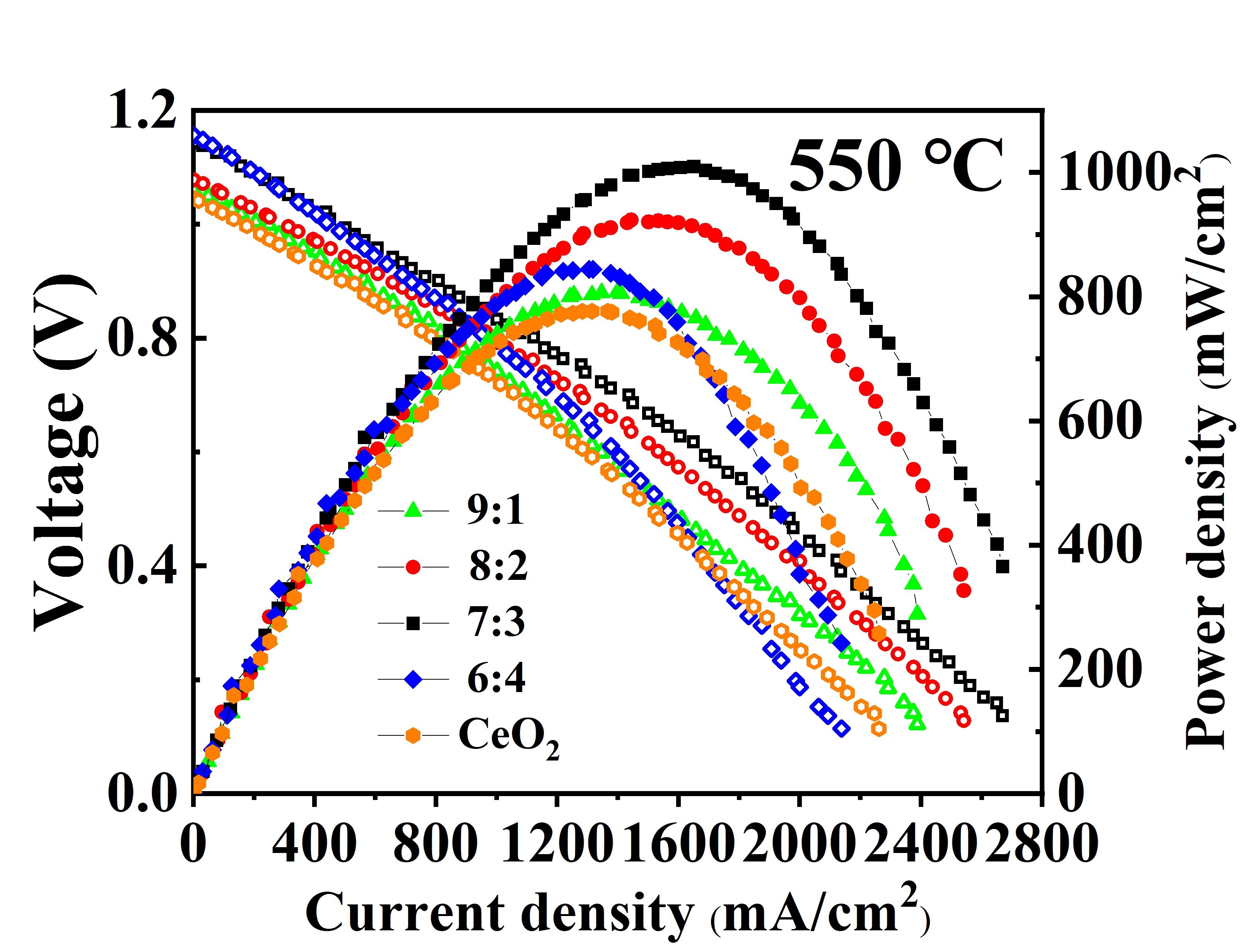


**Figure S4.** IV-IP curves of pure CeO_2_ and CeO_2_/β"-Al_2_O_3_ composites in different metal ions mole ratios at 550 ℃.

SIFC measurements are conducted using different molar ratios of Ce^4+^: Al^3+^ (9:1, 8:2, 7:3, 6:4) of in composite materials to verify the optimal composition. Our research results indicate that the current density/power density is related to the proportion of β"-Al_2_O_3_ introduced, the CeO_2_/β"-Al_2_O_3_ 7:3 fuel cell demonstrates the best results. The findings also showed that all these mass ratios are sufficient to improve OCV of composite electrolyte and prevent the electrical percolation of CeO_2_ particles.

Table S2 Comparison of fuel cell performance between this work and advanced SIFC in the literature.

| Electrolyte | Stability  (h) | HT-MPD (mW·cm^-2^) | LT-MPD (mW·cm^-2^) | Reference |
| --- | --- | --- | --- | --- |
| YSZ/ZnO |  | 550 ℃ (721) | 430 ℃ (142) | [1] |
| ZnO/Sm_2_O_3_ | 40 | 500 ℃ (790) | 450 ℃ (252) | [2] |
| ZnO/MgZnO | 50 | 520 ℃ (997) | 420 ℃ (510) | [3] |
| γ-MnO_2_/GDC |  | 520 ℃ (896) | 440 ℃ (292) | [4] |
| SFT/SnO_2_ |  | 550 ℃ (805) | 370 ℃ (208) | [5] |
| CeO_2_/β"-Al_2_O_3_ | 50 | 550 ℃ (1009) | 350 ℃ (85.9) | This study |

[1] J. Gao, Z. Liu, M. Akbar, et al. Ceramics International, 2023, 49(4): 5637-5645.

[2] H. Wang, E. Hu, F. Wang et al. J. Electrochem. Soc. 2023, 170, 104503.

[3] M.A.K. Yousaf, Y. Lu, M. Naveed, Energy Mater 2022, 2, 200031.

[4] X. Cheng, Y. Yu, J. Huang, et al. International Journal of Hydrogen Energy, 2024, 50, 633-642.

[5] Y. Lu, M.A.K. Yousaf Shah, M. Naveed ACS Appl. Energy Mater. 2023, 6, 12, 6518–6531.

**S5. Surface and cross-sectional SEM images of the surface morphology of BP and AP samples**


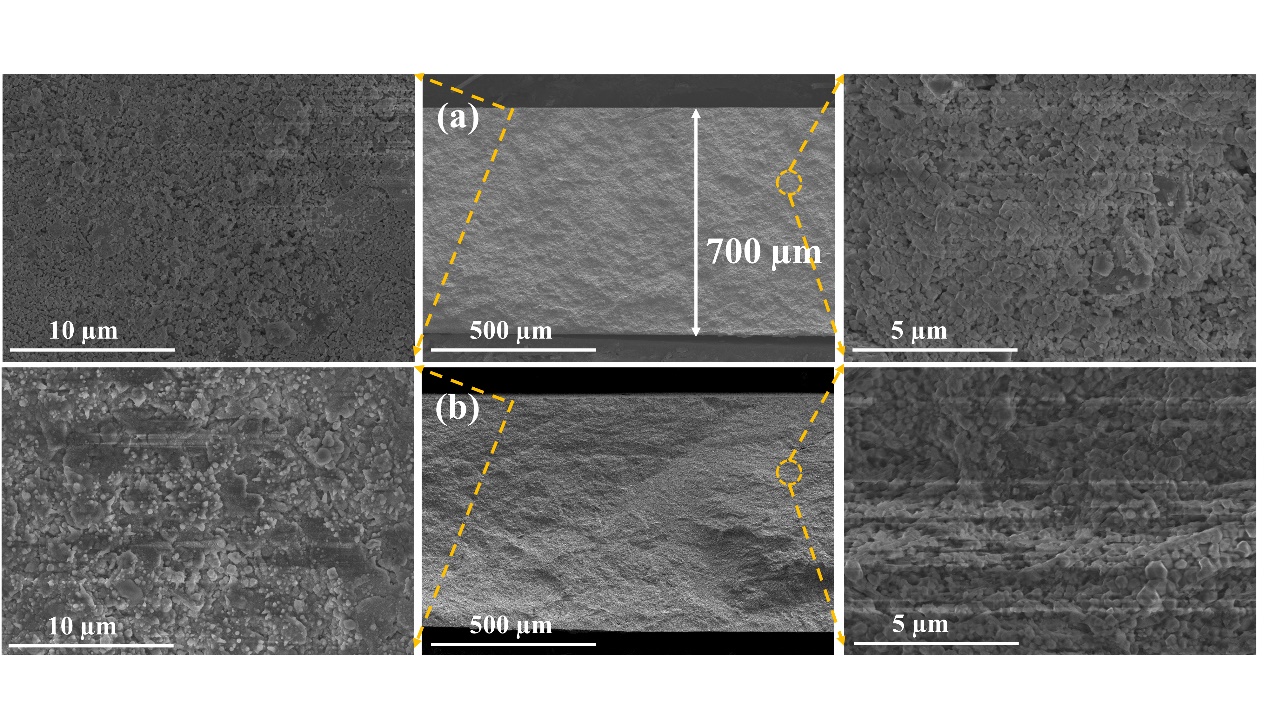


**Figure S5.** Surface and cross-sectional SEM images of the surface morphology of (a) BP and (b) AP samples.

As shown in the figure, the middle layer of the cell was the CeO_2_/β"-Al_2_O_3_ composite electrolyte with a thickness of approximately 0.7 mm. In the surface and cross-sectional SEM image of the AP sample, the dark-gray phase was carbonate mixed in it. The filling of carbonate in the electrolyte results in the coverage of active sites, which hinders the oxygen transport pathway from the electrode to TPB. This greatly increases the mass transfer resistance of oxygen (R_2_), thus, the rapid increase in R_2_ leads to an increase in R_p_, which in turn damages the oxygen reduction reaction (ORR) of the electrode and deteriorates the performance of the SIFCs.

**S6. TG-DSC curves of various combinations of powders**


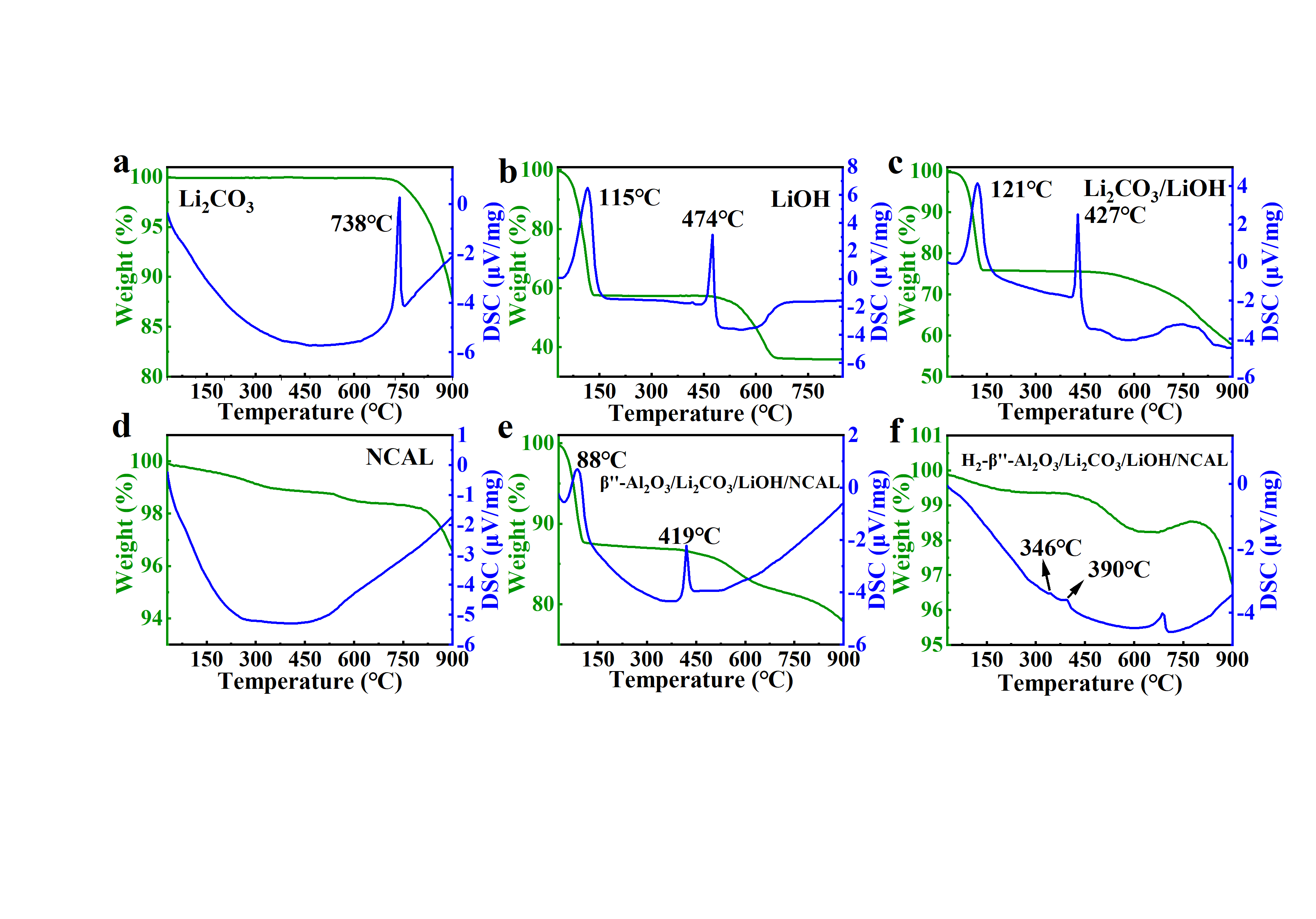


**Figure S6.** TG-DSC curves of various combinations of powders. (a) Li_2_CO_3_ (b)LiOH (c) Li_2_CO_3_/LiOH composite, (d) NCAL (e) β"-Al_2_O_3_/ Li_2_CO_3_/ LiOH/ NCAL composite and (f) the β"-Al_2_O_3_/ Li_2_CO_3_/ LiOH/ NCAL composite treated with H_2_ for 2 hours.

It can be observed that only one composite sample produced an endothermic peak similar to AP powder, Figure S6f shows a melting process from 346 ℃ to 390 ℃ of LiOH, Li_2_CO_3_, β"-Al_2_O_3_, and NCAL composite samples treated with H_2_. Through comparing analysis, it can be determined that the hydroxides/carbonates mixture with reduced melting point originates from the three-phase boundary of the operating fuel cell anode. According to the preview reports, the possible explanation of the decrease of melting temperature is that the wet H_2_ enabled some formation of hydroxides from β"-Al_2_O_3_ with a lower melting point (NaOH 318 ℃, Mg(OH)_2_ 350 ℃). Finally, a mixed state consisting of metal (Li, Na and Mg) hydroxides and carbonates are formed, and its final melting point is shown at 353.5℃.

**Table S3. The impedance fitting results of BP 7:3 and pure CeO_2_ fuel cell**

### Table S3a. The impedance fitting results of BP 7:3 fuel cell. The fitting parameters extracted from ZSimpWin with a equivalent circuit of Ro(R_1_Q_1_)(R_2_Q_2_), where R represents a resistance, and Q is the constant phase element (CPE) representing a non-ideal capacitor. The unit of resistance is Ω·cm^2^, and Q is presented in S Sec^2^ cm^−2^.

| Temperature | R_0_ | R_1_ | Q_1_ | n_1_ | R_2_ | Q_2_ | n_2_ | Chi-Squared |
| --- | --- | --- | --- | --- | --- | --- | --- | --- |
| 550 ℃ | 0.150 | 0.005 | 0.676 | 0.877 | 0.227 | 3.403 | 0.547 | 1.723×10^-4^ |
| 500 ℃ | 0.167 | 0.285 | 2.292 | 0.467 | 0.035 | 2.889 | 1.000 | 8.135×10^-5^ |
| 450 ℃ | 0.202 | 0.010 | 0.498 | 0.882 | 0.367 | 0.674 | 0.614 | 8.552×10^-5^ |
| 400 ℃ | 0.568 | 1.411 | 0.326 | 0.607 | 0.391 | 4.765 | 1.000 | 4.343×10^-5^ |
| 350 ℃ | 0.584 | 0.190 | 0.233 | 0.416 | 31.38 | 0.091 | 0.689 | 1.253×10^-4^ |

### Table S3b. The impedance fitting results of CeO_2_ fuel cell.

| Temperature | R_0_ | R_1_ | Q_1_ | n_1_ | R_2_ | Q_2_ | n_2_ | Chi-Squared |
| --- | --- | --- | --- | --- | --- | --- | --- | --- |
| 550 ℃ | 0.237 | 0.224 | 2.133 | 0.511 | 0.002 | 0.111 | 0.851 | 6.025×10^-5^ |
| 500 ℃ | 0.275 | 0.381 | 1.533 | 0.380 | 0.0003 | 3.94e^-7^ | 9.28e^-4^ | 2.701×10^-4^ |
| 450 ℃ | 0.347 | 0.177 | 2.181 | 0.908 | 0.324 | 0.750 | 0.505 | 1.529×10^-4^ |

**Supplementary references**

[1] G. Zhang, Z. Wen, X. Wu, J. Zhang, G. Ma, J. Jin, *J. Alloys Compd.* **2014,** 613, 80.

[2] G. Kresse,; Furthmuller. J. *Phys. Rev. B* **1996,** 54, 11169.

[3] P. E. Blochl, *Phys. Rev. B* **1994,** 50, 17953.

[4] J. P. Perdew, K. Burke, M.Ernzerhof, *Phys. Rev. B* **1996,** 77, 3865.

[5] H. J. Monkhorst, J. D. Pack, *Phys. Rev. B* **1976,** 13, 5188.

[6] S. L. Dudarev, G. A. Botton, S. Y. Savrasov, C. J. Humphreys, A. P. Sutton, *Phys. Rev. B* **1998,** 57, 1505.

[7] G. Henkelman, B. P. Uberuaga, H. Jónsson, *J. Chem. Phys.* **2000,** 113, 9901.
